# Buildings Contemplated

**Published:** 1914-07-25

**Authors:** 


### Buildings Contemplated.

Carnarvon.—The surveyors' plans for a new hospital on the site of Cynifryn have been formally approved by the Town Council.

Durham.—Messrs. Newcombe and Newcombe, of Newcastle-upon-Tyne, are the architects for the proposed extensions to the Durham County Hospital, which will involve a considerable expenditure.

Ferry Fryston.—The Doncaster and Sheffield Guardians have agreed conjointly to purchase Fryston Hall, at a cost of £10,000, to be used as a home for the feeble-minded.

Summersdale. — The Westhampnett Rural District Council have instructed their surveyor, Mr. L. Bell, to prepare plans and estimates for an isolation hospital at Summersdale.

West Sussex.—The West Sussex County Council has under consideration a scheme for the treatment of cases of fever and tuberculosis, which is to apply to the whole of West Sussex, with the exception of Worthing, Chichester, and Bognor, involving an outlay of £36.000.

